# Triglyceride-glucose index and the incidence of stroke: A meta-analysis of cohort studies

**DOI:** 10.3389/fneur.2022.1033385

**Published:** 2023-01-04

**Authors:** Canlin Liao, Haixiong Xu, Tao Jin, Ke Xu, Zhennan Xu, Lingzhen Zhu, Mingfa Liu

**Affiliations:** Department of Neurosurgery, Shantou Central Hospital, Shantou, Guangdong, China

**Keywords:** triglyceride-glucose index, insulin resistance, stroke, meta-analysis triglyceride-glucose index, meta-analysis

## Abstract

**Background:**

Insulin resistance (IR) is involved in the pathogenesis of atherosclerosis. As a new indicator, the triglyceride-glucose (TyG) index has greater operability for the evaluation of insulin resistance. Previous studies have shown inconsistent results in evaluating the association between the TyG index and stroke incidence in people without stroke at baseline. Therefore, this study aimed to systematically assess this association through a meta-analysis.

**Methods:**

Cohort studies with the multivariate-adjusted hazard ratio (HR) association between the TyG index and stroke were obtained by searching the PubMed, Cochrane Library, and EMBASE databases before 16 December 2021. We pooled the adjusted HR along with 95% CI using a random-effects model. The primary outcome was stroke including ischemic and hemorrhagic stroke. We conducted subgroup analyses stratified by study design, ethnicity, characteristics of participants, weight of studies, and length of follow-up duration. Review Manager 5.3 and Stata 17 were used to perform the meta-analysis.

**Results:**

Eight cohort studies with 5,804,215 participants were included. The results showed that participants with the highest TyG index category at baseline compared to those with the lowest TyG index category were independently associated with a higher risk of stroke (HR: 1.26, 95% CI: 1.24–1.29, I^2^ = 0%, *P* < 0.001). This finding was consistent with the results of the meta-analysis with the TyG index analyzed as a continuous variable (HR per each-unit increment of the TyG index: 1.13, 95% CI 1.09–1.18, I^2^ = 0%, *P* < 0.001). Subgroup analysis had no significant effects (for subgroup analysis, all *P* > 0.05). No significant heterogeneity was observed among the included cohort studies.

**Conclusion:**

A higher TyG index may be independently associated with a higher risk of stroke in individuals without stroke at baseline. The aforementioned findings need to be verified by a large-scale prospective cohort study to further clarify the underlying pathophysiological mechanism between the TyG index and stroke.

## 1. Introduction

Stroke is one of the most devastating diseases in the world. Globally, it is the second leading cause of the increase in years of life lost (1). In addition, the increasingly youthful trend of stroke deserves our great attention (2). Ischemic stroke is the result of blood circulation disorders in the cerebral blood vessels caused by occlusion of the large cerebral arteries, which occurs more commonly in the middle cerebral artery (3) or cerebral small vessel disease (4). Previous studies have demonstrated that insulin resistance plays an important role in the pathogenesis of ischemic stroke (5).

The hyperinsulinemic–euglycemic clamp test (HIEC) is the gold standard for assessing insulin resistance. Due to the complexity of the test process, the extensive time required, and the high cost, its clinical application is very limited (6). The homeostasis model assessment of insulin resistance (HOMA-IR) index is not very convenient and economical in clinical application, although it is the most accessible indicator for evaluating insulin resistance in clinical practice (7).

As a novel surrogate indicator of insulin resistance, the triglyceride-glucose (TyG) index, derived from the fasting triglyceride and glucose levels, is convenient and quick to obtain, economical, and reliable (8). The TyG index can be calculated as follows: ln [triglyceride level (mg/dL) ×fasting blood glucose level (mg/dL)/2] (9, 10). Studies have confirmed that the TyG index is significantly correlated with both HIEC and HOMA-IR (11). Therefore, the TyG index can be used as an easily accessible and operational index of insulin resistance.

Observational studies have revealed a relationship between a high TyG index and stroke in their populations. However, most of them were cross-sectional studies (12, 13). Recently, as an increasing number of cohort studies on stroke and the TyG index have been published, we have found inconsistent results (14–17). Therefore, our study aimed to summarize the association between the baseline TyG index and stroke incidence in patients without stroke at baseline.

## 2. Methods

This meta-analysis was based on the Preferred Reporting Items for Systematic Reviews and Meta-Analysis (18) (http://www.prisma-statement.org/) and Cochrane Handbook (19, 20). Electronic databases including PubMed, the Cochrane Library (CENTRAL), and EMBASE were searched for relevant studies and literature.

### 2.1. Study selection

Studies adhering to all the following criteria were included: (1) Participants were adults with no stroke at baseline; (2) cohort studies were published as full-length articles in English; (3) the TyG index was measured at baseline; (4) the outcome included the occurrence of a stroke or ischemic stroke; (5) risk factors adjusted for potential confounders were reported; and (6) hazard ratios (HRs) were reported. In contrast, studies were excluded from the meta-analysis if they met at least one of the following criteria: (1) participants were <18 years of age; (2) the studies were not cohort studies; (3) there was no reporting of stroke; (4) there was no measurement of the TyG index; (5) reported data were based on univariate analysis rather than multivariate analysis; and (6) HRs were not reported.

Two researchers (CL and KX) used the PICOS principles to search for related literature and independently evaluated the literature. Disputes were resolved after a discussion with a third researcher (LZ).

### 2.2. Data extraction

Two researchers (CL and KX) independently extracted data from the articles. The extracted content included the names of the authors, publication year, study design, country, participant characteristics, average age, proportion of male participants, proportion of patients with diabetes, TyG index analysis, follow-up duration, and result validation. After data extraction, the two researchers exchanged data for verification.

### 2.3. Literature search

The PubMed, Cochrane Library (CENTRAL), and EMBASE databases were searched using a combination of the following terms: (1) “triglyceride and glucose index” OR “triglyceride-glucose index ^*^” OR “TyG index” OR “triglyceride glucose index” OR “triacylglycerol glucose index”; (2) “stroke” OR “Cerebrovascular Accident” OR “Cerebrovascular Accidents” OR “CVA” OR CVAs; OR “Apoplexy” OR “Brain Vascular Accident” OR “Brain Vascular Accidents” (Supplementary Table S1). Reference lists of original and review articles that are related were manually searched for potentially eligible studies. The final literature search was conducted on 16 December 2021.

### 2.3. Literature screening

The search results obtained from the PubMed, Cochrane Library (CENTRAL), and EMBASE databases were exported to Endnote X9, whose function of “duplicate finder” was used to identify and remove repetitive literature. Literature screening was divided into two stages. First, we conducted a preliminary screening based on the titles and abstracts of the literature to obtain possibly eligible, eligibility-unknown, and clearly eligible literature. For literature that might be eligible and those whose eligibility was unknown, their full-length texts were obtained and further selected according to the inclusion and exclusion criteria, thus obtaining eligible studies. Titles, abstracts, and full-length texts were selected by two researchers (ZX and LZ), strictly and independently, based on the inclusion and exclusion criteria. When the screening results were inconsistent, the two researchers discussed and negotiated with each other to reach a consensus. If the negotiation failed, we consulted a third researcher (TJ) and adopted his opinion.

#### 2.3.1. Quality evaluation

The Newcastle–Ottawa Scale (20) was used to evaluate the quality of each study according to the selection of the study groups, comparability of the groups, and ascertainment of the outcome of interest. The scale ranges from 1 to 9, and studies with test results of more than six are classified as high quality. The assessment was performed independently by two researchers (LZ and ZX). Any disagreement between researchers was resolved by consensus. If the negotiation failed, we consulted a third researcher (TJ) and adopted his opinion.

#### 2.3.2. Data analyses

Hazard ratios and their corresponding 95% confidence intervals (CIs) were used as a general measure of the association between the TyG index and stroke in people who had no stroke at the baseline examination. For the study that analyzed the TyG index as a categorical variable, the HRs of the incidence of stroke in participants with the highest TyG index level compared to those with the lowest TyG index level were extracted. For studies where the TyG index was analyzed as a continuous variable, the HRs of stroke incidence were extracted for each-unit increment of the TyG index. The Cochran *Q*-test and I^2^ estimation were used to assess the heterogeneity of the included cohort studies (21). If I^2^ was <50%, it was considered that there was no significant heterogeneity. In addition, a random-effect model was used to synthesize HRs data, as this model was considered a more general method that could incorporate potential heterogeneity into the study (19). Furthermore, sensitivity analyses, excluding one individual study at a time, were conducted to test the stability of the results (22). Predefined subgroup analyses were also performed to evaluate the impact of study characteristics, including study design, participant characteristics, participant ethnicity, weight of studies, and follow-up duration on the association between the TyG index and stroke incidence. All studies included adjusted variables. The baseline TyG index was analyzed as categorical variables The median, quartile, or quintile was used to divide the research participants into a higher TyG index group and a lower TyG index group. After adjusting for variables, the HRs and 95% CIs of stroke or ischemic stroke were calculated in the higher TyG index group during the follow-up period, with the lowest TyG index group as a reference. Potential publication bias was assessed by visual inspection of the funnel plot symmetry. Review Manager (version 5.3; Cochrane Collaboration, Oxford, UK) and Stata 17 (Stata Corp., College Station, Texas, USA) were used to perform the statistical analyses.

## 3. Results

### 3.1. Process and results of the literature screening

**Figure 1:**
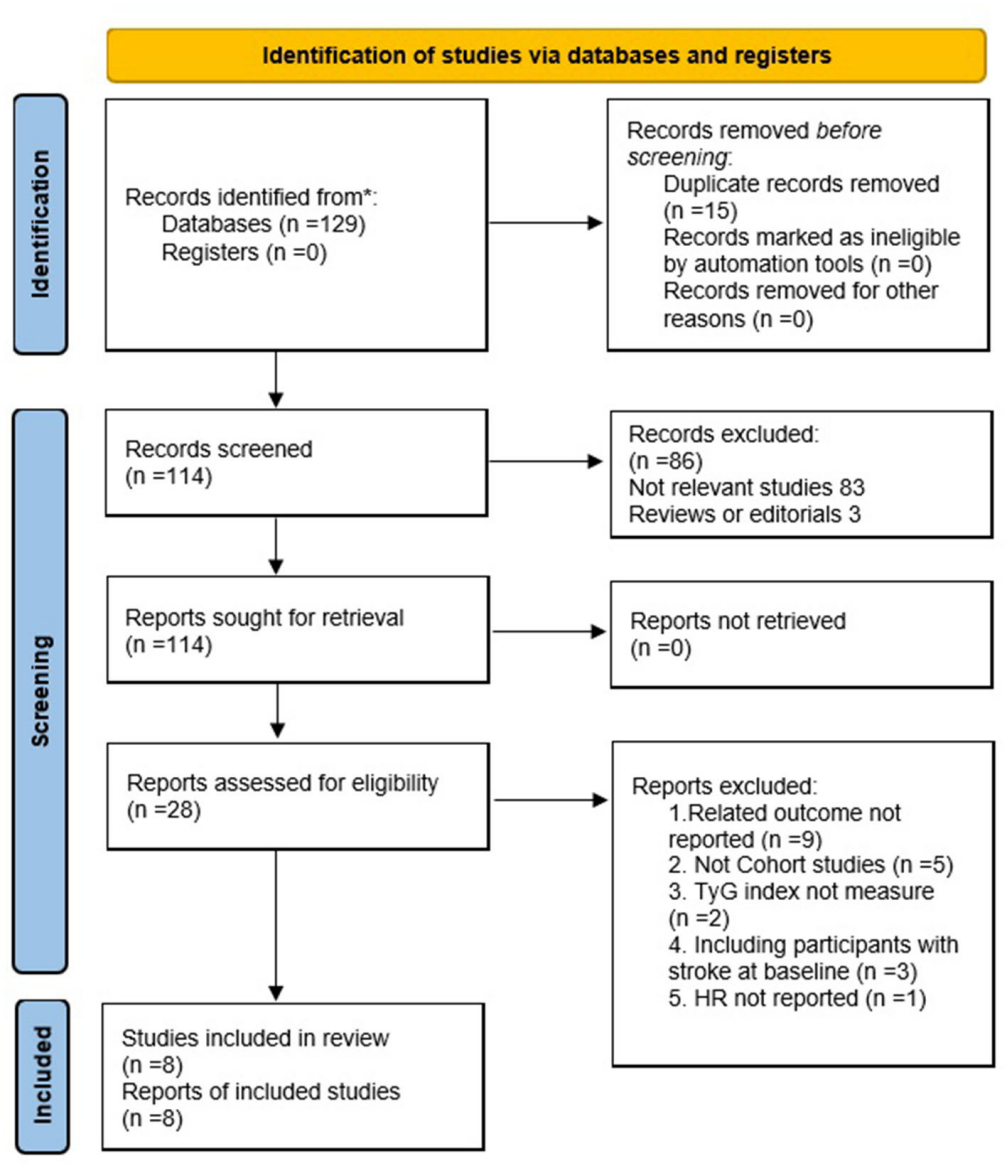
Flowchart of the databases search and study identification.

The search strategy retrieved 129 articles through PubMed, Cochrane Library (CENTRAL), and EMBASE databases (Figure 1). A total of 114 articles were obtained after excluding 15 duplications. Eight studies comprising 5,804,215 participants were included in the meta-analysis after further evaluation of the abstract and full-length text twice, according to the inclusion criteria.

### 3.2. Study characteristics and quality evaluation

#### 3.2.1. Study characteristics

The characteristics of the eight cohort studies (14–17), included the name of the author(s), publication year, study design, country, participant characteristics, number of participants, average age of participant, proportion of men, proportion of patients with diabetes, TyG index analysis, follow-up duration, result verification, outcome reported, and adjusted variables (Table 1). Overall, eight cohort studies with 5,804,215 participants were included. Four out of the eight were prospective cohort studies (16, 17, 25, 26), and the remaining four were retrospective cohort studies (14, 15, 23, 26). The research participants of four studies were participants without stroke in the community (14, 16, 23, 24), while those of the other studies were outpatients or inpatients in hospitals (15, 17, 25, 26). The studies were performed in China (14–16, 24–26), South Korea (23), and Spain (17). These studies were published from 2016 to 2021, where patients at baseline were followed up for time ranging from post-intervention to 11.02 years. Five studies (14, 16, 17, 23, 24) were followed for more than 5 years and three studies (15, 25, 26) for less than 5 years. The two articles produced by the Kailuan study provided different variables, with one for categorical (16) and the other for continuous (24).

**Table 1:** Characteristics of the included cohort studies.

**Study**	**Year**	**Design**	**Country**	**Characteristics of participants**	**Number of participants**	**Mean age (Years)**	**Male (%)**	**Proportion of DM**	**TyG index analysis**	**Follow-up duration (years)**	**Outcome validation**	**Outcomes reported**	**Variables** **adjusted**
Sanchez-Inigo et al. (17)	2016	PC	Spain	First-time attendee outpatients to an internal medicine department without ASCVDs	5,014	54.4	61.2	5.2	Q5:Q1	8.8	ICD-10	stroke (157)	Age, sex, BMI, smoking, alcohol intake, lifestyle pattern, HTN, T2DM, antiplatelet, therapy, HDL-C, and LDL-C
Li et al. (14)	2019	RC	China	Participants aged over 60 years without stroke who participated in a routine health check-up program	6,078	70.5	53.1	11.8	Q4: Q1	5.5	ICD-10	stroke (234)	Age, sex, living, alone, current, smoker, alcohol, consumption, exercise, BMI, SBP, HDL-C, LDLC, and T2DM
Mao et al. (25)	2019	PC	China	patients diagnosed with NSTE-ACS without stroke	791	62.5	67.4	32.6	M2:M1	1	Clinical evaluation	Stroke (5)	Age, sex, metabolic syndrome, LDL-C, HDL-C, SYNTAX score, CRP, basal insulin, sulfonylurea, metformin, α-glucosidase inhibitor, ACEI/ARB, beta-blocker, and PCI/CABG.
Hong et al. (23)	2020	RC	Korea	Community population without stroke	5,593,134	53.0	50.5	3.7	Q4:Q1	8.2	ICD-10	Stroke (89,120)	Age, sex, smoking, alcohol, consumption, regular physical activity, low socioeconomic, status, BMI, HTN, and TC
Wang et al. (16)	2020	RC	China	consecutive patients with diabetes who underwent coronary angiography for ACS	3,428	66.3	55.9	100	T3:T1	3	Clinical evaluation	non-fatal stroke (46)	Age, male, smoker, previous MI, previous CABG, BMI, AMI, LVEF, left main disease, multi-vessel disease, HbA1c, hs-CRP, statin, insulin
Zhao et al. (27)	2020	RC	China	patients with NSTE-ACS, who received elective PCI without diabetes	1,576	59.7	73.7	0	M2:M1	2	Clinical evaluation	non-fatal ischemic stroke (27)	Age, gender, smoking history, hypertension, dyslipidemia, previous history of MI, PCI, stroke and PAD, eGFR, LVEF, LM disease, three-vessel disease, SYNTAX score, number of stents, statins at discharge and ACEI/ARB at discharge ACEI/ARB
Wang et al. (16)	2021	PC	China	Community population without stroke	97,653	51.67	79.62	2.93	Q4:Q1	11.02	Clinical evaluation	Stroke (5122) ischemic stroke (4277)	Age, gender, level of education, income, smoking, alcohol abuse, physical activity, BMI, SBP, DBP, history of MI, dyslipidemia, HDL-C, LDL-C, Hs-CRP, antidiabetic drugs, lipid-lowering drugs, HTN, DM, antihypertensive drugs
Liu et al. (24)	2021	PC	China	Community population without stroke	96,541	51.19	79.61	9.06	Q4:Q1	10.33	Clinical evaluation	Stroke (5083) ischemic stroke (4266) Ischemic stroke (677) Hemorrhagic stroke (1024)	Age, gender, current smoking status, current drinking status, physical activity, education, BMI, hypertension, diabetes, HDL-C, LDL-C, Hs-CRP, lipid-lowering medication, antidiabetic medication, and antihypertensive medication. Age, gender; marital status, income, education level, smoking, alcohol drinking, physical activity, family history of stroke, SBP, DBP, resting heart rate, BMI, WC, TC, HDL-C and LDL-C.

TyG, triglyceride–glucose index; PC, prospective cohort; RC, retrospective cohort; Q5:Q1, the 5th quintile vs. the 1st quintile; Q4:Q1, the 4th quartile vs. the 1st quartile; T3:T1, the 3rd tertile vs. the 1st tertile; M2:M1, the 2nd median vs. the 1st median; T2DM, type 2 diabetes mellitus; ICD-10, International Classification of Diseases, tenth edition; PAD, peripheral artery disease; HTN, hypertension; HDL-C, high-density lipoprotein cholesterol; LDL-C, low-density lipoprotein cholesterol; BMI, body mass index; WC, wrist circumference; eGFR, estimated glomerular filtrating rate; SBP, systolic blood pressure; DBP, diastolic blood pressure; TC, total cholesterol; hs-CRP: high-sensitivity C-reactive protein; CABG, coronary artery bypass grafting; PCI, percutaneous transluminal coronary intervention; AMI, acute myocardial infarction; MI, myocardial infarction; LVEF, left ventricular ejection fraction.

#### 3.2.2. Quality evaluation

Eight studies included in this meta-analysis were cohort studies. The Newcastle–Ottawa Scale (20) was used to evaluate their quality, and the results showed that three studies scored seven points and the other five studies scored nine points. All included cohort studies were judged high quality (Table 2).

**Table 2:** Details of quality evaluation *via* the Newcastle–Ottawa Scale.

**Study (Publication Year)**	**Selection of cohorts**			**Comparability of cohorts**			**Outcome of cohorts**			**Total**
	**a**	**b**	**c**	**d**	**e**	**f**	**g**	**h**	**i**	
Sanchez-Inigo et al. (17)	1	1	1	1	1	1	1	1	1	9
Li et al. (14)	1	1	1	1	1	1	1	1	1	9
Hong et al. (23)	1	1	1	1	1	1	1	1	1	9
Mao et al. (25)	1	1	1	1	1	1	1	0	0	7
Wang et al. (16)	1	1	1	1	1	1	1	0	0	7
Wang et al. (16)	1	1	1	1	1	1	1	1	1	9
Zhao et al. (15)	1	1	1	1	1	1	1	0	0	7
Liu et al. (24)	1	1	1	1	1	1	1	1	1	9

a. Representativeness of the exposed cohort. b. Selection of the non-exposed cohort. c. Ascertainment of exposure. d. Demonstration that outcome of interest was not present at start of study. e. Comparability of cohorts on the basis of the design or analysis (adjusted for age and gender). f. Comparability of cohorts on the basis of the design or analysis (adjusted for any other factor). g. Assessment of outcome. h. Was follow-up long enough for outcomes to occur (>5 years). i. Adequacy of follow-up of cohorts (>5 years). The scale ranges from one to nine in total, and judge studies above six as high-quality cohort studies.

#### 3.2.3. Results of the meta-analysis of the cohort studies

**Figure 2:**
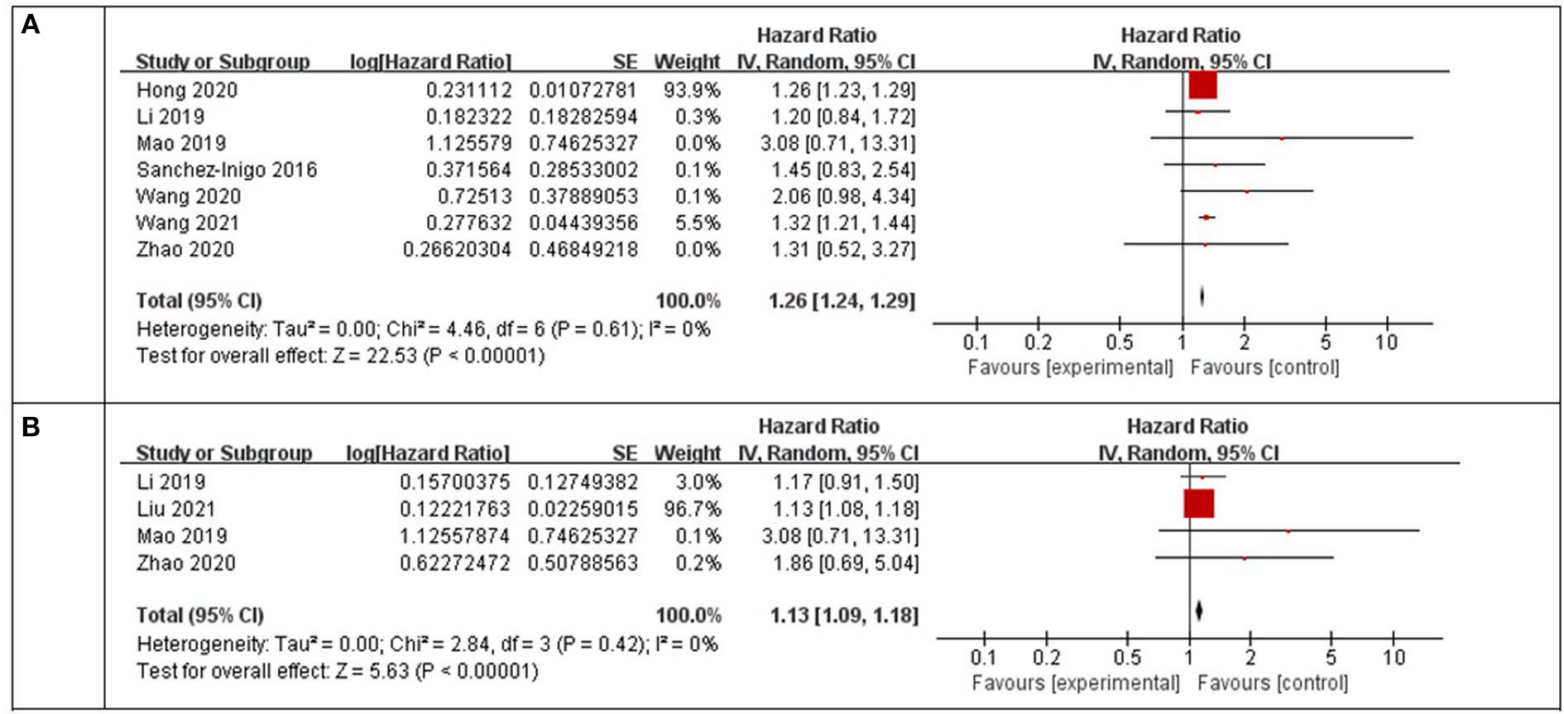
Forest plots for the meta-analysis of the association between the TyG index and the risk of stroke. **(A)** Meta-analysis with the TyG index analyzed as a categorical variable. **(B)** Meta-analysis with the TyG index analyzed as a continuous variable.

**Figure 3:**
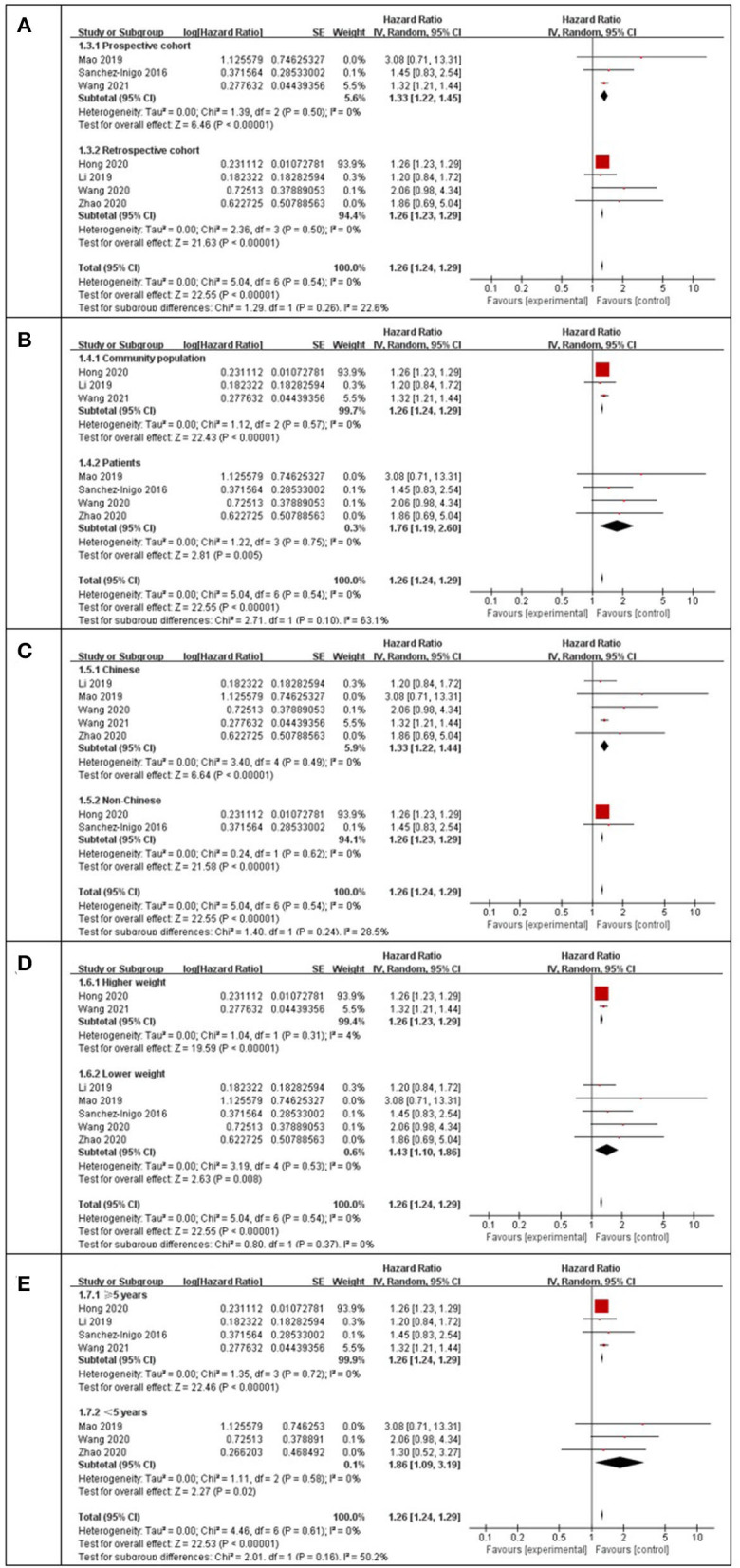
Subgroup analysis for the Meta-analysis of association between the TyG index and the risk of stroke. **(A)** Subgroup analysis according to study design. **(B)** Subgroup analysis according to characteristics of participants. **(C)** Subgroup analysis according to the ethnicity of the population. **(D)** Subgroup analysis according to the weight of studies. **(E)** Subgroup analysis according to the length of follow-up duration.

Using a random-effects model, the pooled results of seven cohort studies (14–17, 23, 25–27) showed that compared to participants with the lowest TyG index category at baseline, those with the highest TyG index category had a significantly increased incidence of stroke during the follow-up (HR: 1.26, 95% CI: 1.24–1.29, I^2^ = 0%, *P* < 0.001; Figure 2A). This finding was consistent with the TyG index analyzed as a continuous variable (four studies, HR per each-unit increment of the TyG index: 1.13, 95% CI 1.09–1.18, I^2^ = 0%, *P* < 0.001; Figure 2B). Subgroup analyses showed a consistent association between the prospective studies (HR: 1.33, 95% CI: 1.22–1.45, I^2^ = 0%, *P* < 0.001; Figure 3A) and retrospective studies (HR: 1.26, 95% CI: 1.23–1.29, I^2^ = 0%, *P* < 0.001; Figure 3A); the community population (HR: 1.26, 95% CI: 1.24–1.29, I^2^ = 0%, *P* < 0.001; Figure 3B) and outpatient or inpatient populations (HR: 1.76, 95% CI: 1.19–2.60, I^2^ = 0%, *P* = 0.005; Figure 3B); Chinese (HR: 1.33, 95% CI: 1.22–1.44, I^2^ = 0%, *P* < 0.001; Figure 3C), non-Chinese participants (HR: 1.26, 95% CI: 1.23–1.29, I^2^ = 0%, *P* < 0.001; Figure 3C); higher weight (HR: 1.26, 95% CI: 1.23–1.29, I^2^ = 4%, *P* < 0.001; Figure 3D) and lower weight (HR: 1.43, 95% CI: 1.10–1.86, I^2^ = 0%, *P* = 0.008; Figure 3D); follow-up duration more than 5 years (HR: 1.26, 95% CI: 1.24–1.29, I^2^ = 0%, *P* < 0.001; Figure 3E) and less than 5 years (HR: 1.86, 95% CI: 1.09–3.19, I^2^ = 0%, *P* = 0.02; Figure 3E). The leave-one-out analysis showed similar results (Supplementary Figure S1).

#### 3.2.4. Publication bias

**Figure 4:**
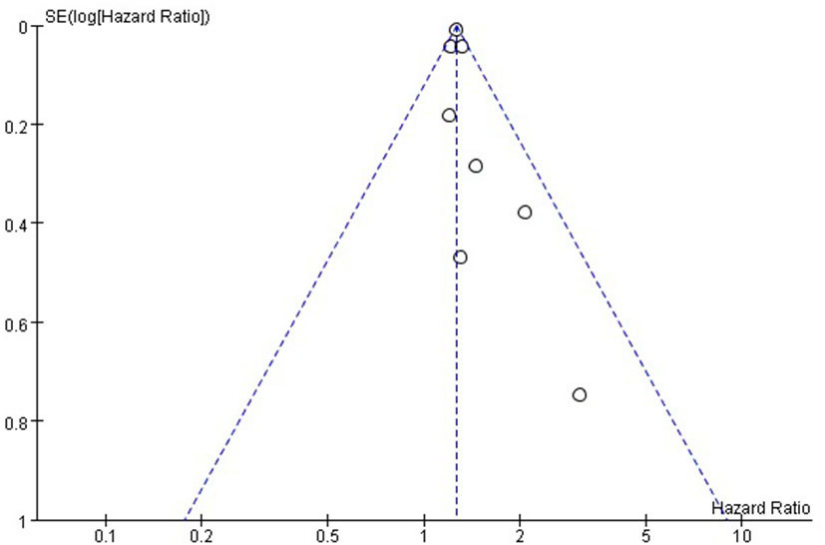
Funnel plot for the publication bias underlying the metaanalysis of the associtation between TyG index and stroke.

Funnel plots were drawn using stroke as an outcome indicator to observe publication bias in the eight cohort studies. Funnel plots were symmetric on visual inspection, suggesting a low risk of publication bias (Figure 4). As only eight studies (14–17, 23–26) were included, < 10 studies were required, and the Egger regression test could not be performed in this study (28).

## 4. Discussion

This meta-analysis of cohort studies showed that a higher TyG index at baseline was independently associated with an increased incidence of stroke regardless of whether the TyG index was analyzed as a categorical or continuous variable. Moreover, consistent results were obtained in subgroup analysis according to the study design, ethnicity, characteristics of participants, weight of studies, and length of follow-up duration. These results suggest that a higher TyG index may be an independent predictor of increased stroke incidence in the general adult population without stroke at baseline.

Our meta-analysis has some advantages and is included below. First, only cohort studies were included; thus, potential recall bias associated with the cross-sectional design was avoided. In addition, in order to have a more accurate statistical description and significance for cohort studies, we included only studies with multivariate-adjusted HR, which not only avoids potential confounding biases but also provides an independent association between the TyG index and stroke. Moreover, all the included studies are high-quality cohort studies with large numbers of participants. Otherwise, sensitivity and subgroup analyses were performed for all included studies to ensure the robustness of the results. Finally, all the I^2^ in the meta-analysis were lower than in previous studies, and no significant heterogeneity was observed among the included cohort studies. Our meta-analysis demonstrated the association between the TyG index and the increased incidence of stroke which indicates underlying pathophysiological mechanisms between insulin resistance and stroke exists. Insulin resistance not only enhances the adhesion, activation, and aggregation of platelets, but it also causes hemodynamic disturbances, all of which are conducive to the occurrence of ischemic stroke (5). In addition, it can cause an imbalance in glucose metabolism, leading to chronic hyperglycemia. This, in turn, triggers oxidative stress and inflammation, leading to cell damage and atherosclerotic plaque formation (29).

The TyG index, as a result of triglycerides and fasting blood glucose, has been recognized as a simple and reliable surrogate indicator of insulin resistance (30). In clinical applications, it is economical to measure blood triglycerides and fasting blood glucose, and the TyG index can be obtained through simple calculations. A previous study proved that the TyG index has high sensitivity and specificity in detecting insulin resistance (10), and it is superior to HOMA-IR (31). Moreover, compared with HOMA-IR, the TyG index, which does not require measurement of insulin levels, can be conveniently and economically used for all patients and healthy people and is also suitable for large-scale screening of insulin resistance. However, further studies are needed to conduct whether the TyG index could be added to stroke prediction tools such as the Framingham Stroke Risk Profile (32) and measure the critical value of the TyG index in the general adult population.

When the results of the meta-analysis are interpreted, some limitations should be observed. First, in the subgroup analysis, only the study design, participant characteristics, participant ethnicity, weight of studies, and follow-up duration were analyzed. More research is needed to determine whether other research characteristics will affect the results, such as sex, diabetes status, and concurrent medications used. Third, among the studies we eventually included, there were six Chinese studies and only two non-Chinese studies, one from Asia and the other from Europe. Data from other countries such as the United States, Australia, and Africa are still scarce, thus, a more detailed ethnic subgroup analysis should be conducted. Fourth, owing to the limitations of the research data, hemorrhagic stroke cannot be evaluated in a systematic manner. Fifth, although the cohort studies included were all adjusted for in the multivariate analysis, the influence of unadjusted participating factors in the cohort studies could not be ruled out based on the HR of the study and the association between the TyG index and the incidence of stroke. Similarly, we do not know whether the data before the multivariate adjustment had an impact on the study. Finally, even though we conducted a subgroup analysis, we found a significant effect after excluding two larger studies (16, 23), which had a combined weight of 99.4% and had a major influence on the meta-analysis.

## 5. Conclusion

A higher TyG index may be independently associated with a higher risk of stroke in individuals without stroke at baseline. The aforementioned findings need to be verified by a large-scale prospective cohort study to further clarify the underlying pathophysiological mechanism between the TyG index and stroke.

## Data availability statement

The raw data supporting the conclusions of this article will be made available by the authors, without undue reservation.

## Author contributions

CL, KX, and LZ conceived, designed the research, performed the literature search, and data extraction. ZX, LZ, and TJ performed the literature screening and quality evaluation. CL and KX analyze data and wrote the initial manuscript. HX and ML revised the manuscript. ML had primary responsibility for the final content. All authors reviewed, revised, and approved the final manuscript for submission.
