# Supplementary material for: Triglyceride-glucose index and the incidence of stroke: A meta-analysis of cohort studies
Source: Front Neurol. 2023 Jan 4;13:1033385. doi: 10.3389/fneur.2022.1033385 (PMC9845890; doi:10.3389/fneur.2022.1033385)
Supplement: Supplementary file 1 [file Data_Sheet_1.ZIP › PRISMA_2020_flow_diagram_new_SRs_v1.docx]

**Identification of studies via databases and registers**

Records removed *before screening*:

Duplicate records removed

(n =15)

Records marked as ineligible by automation tools (n =0)

Records removed for other reasons (n =0)

Records identified from*:

Databases (n =129)

Registers (n =0)

**Identification**

Records excluded:

(n =86)

Not relevant studies 83

Reviews or editorials 3

Records screened

(n =114)

Reports sought for retrieval

(n =114)

Reports not retrieved

(n =0)

**Screening**

Reports excluded:

1.Related outcome not reported (n =9)

2. Not Cohort studies (n =5)

3. TyG index not measure

(n =2)

4. Including participants with stroke at baseline (n =3)

5. HR not reported (n =1)

Reports assessed for eligibility

(n =28)

Studies included in review

(n =8)

Reports of included studies

(n =8)

**Included**

*Consider, if feasible to do so, reporting the number of records identified from each database or register searched (rather than the total number across all databases/registers).

**If automation tools were used, indicate how many records were excluded by a human and how many were excluded by automation tools.

*From:*  Page MJ, McKenzie JE, Bossuyt PM, Boutron I, Hoffmann TC, Mulrow CD, et al. The PRISMA 2020 statement: an updated guideline for reporting systematic reviews. BMJ 2021;372:n71. doi: 10.1136/bmj.n71

For more information, visit: <http://www.prisma-statement.org/>
