# Supplementary material for: Triglyceride-glucose index and the incidence of stroke: A meta-analysis of cohort studies
Source: Front Neurol. 2023 Jan 4;13:1033385. doi: 10.3389/fneur.2022.1033385 (PMC9845890; doi:10.3389/fneur.2022.1033385)

**Table S1:** Detailed description of the search strategy

| **PubMed** | |
| --- | --- |
| #1 | ‘triglyceride-glucose index’[MeSH Terms] |
| #2 | ‘triglyceride and glucose index’ OR ‘triglyceride-glucose index *’ OR ‘TyG index’ OR ‘triglyceride glucose index’ OR ‘triacylglycerol glucose index’ |
| #3 | ‘Stroke’[MeSH Terms] |
| #4 | ‘stroke’ OR ‘Cerebrovascular Accident’ OR ‘Cerebrovascular Accidents’ OR ‘CVA’ OR ‘CVAs’ OR ‘Apoplexy’ OR ‘Brain Vascular Accident’ OR ‘Brain Vascular Accidents’ OR ‘Brain Vascular Accident’ |
| #5 | #1 OR #2 |
| #6 | ‘#3 OR #4 |
| #7 | #5 AND #6 |
| **Embase** | |
| #1 | ‘triglyceride-glucose index’: ab,ti |
| #2 | ‘triglyceride and glucose index’ OR ‘triglyceride-glucose index *’ OR ‘TyG index’ OR ‘triglyceride glucose index’ OR ‘triacylglycerol glucose index’ |
| #3 | ‘Stroke’: ab,ti |
| #4 | ‘stroke’ OR ‘Cerebrovascular Accident’ OR ‘Cerebrovascular Accidents’ OR ‘CVA’ OR ‘CVAs’ OR ‘Apoplexy’ OR ‘Brain Vascular Accident’ OR ‘Brain Vascular Accidents’ OR ‘Brain Vascular Accident’ |
| #5 | #1 OR #2 |
| #6 | #3 OR #4 |
| #7 | #5 AND #6 |
| **Cochrane** | |
| #1 | ‘triglyceride-glucose index’:ti, ab, kw |
| #2 | ‘triglyceride and glucose index’ OR ‘triglyceride-glucose index *’ OR ‘TyG index’ OR ‘triglyceride glucose index’ OR ‘triacylglycerol glucose index’ |
| #3 | ‘Stroke’:ti,ab,kw |
| #4 | ‘stroke’ OR ‘Cerebrovascular Accident’ OR ‘Cerebrovascular Accidents’ OR ‘CVA’ OR ‘CVAs’ OR ‘Apoplexy’ OR ‘Brain Vascular Accident’ OR ‘Brain Vascular Accidents’ OR ‘Brain Vascular Accident’ |
| #5 | #1 OR #2 |
| #6 | #3 OR #4 |
| #7 | #5 AND #6 |

**Table S2:** Studies excluded (n=20) with reasons

| Studies excluded | Reasons |
| --- | --- |
| Zhao,Y 2021[1] | Not target outcome: Using Risk Ratio instead of Hazard Ratio |
| Zhao,X 2021[2] | Not target outcome: Multivariable adjusted HR for stroke |
| Wang,A 2021[3] | Not target outcome: baseline TyG Index |
| Nam Ki-Woong 2021[4] | Cross-sectional study |
| Nam Ki-Woong 2021[5] | Including participants with stroke at baseline |
| Lee,M 2021[6] | Including participants with stroke at baseline |
| Hou,Z 2021[7] | Not cohort study |
| Chang,Y 2021[8] | TyG index not measure |
| Zhou,Y 2020[9] | Including participants with stroke at baseline |
| Zhang,B 2020[10] | Cross-sectional study |
| Shi,W 2020[11] | Cross-sectional study |
| Ma,X 2020[12] | Not target outcome: Multivariable adjusted HR for stroke |
| Chiu,H 2020[13] | Not target outcome: Multivariable adjusted HR for stroke |
| Su,W 2019[14] | Not target outcome: Multivariable adjusted HR for stroke |
| Li,R 2019 [15] | Cross-sectional study |
| Won,K 2018[16] | Not target outcome: Multivariable adjusted HR for stroke |
| Won,K 2018[17] | Not target outcome: Multivariable adjusted HR for stroke |
| Jin,J 2018[18] | Not target outcome: Multivariable adjusted HR for stroke |
| Sánchez-Iñigo 2017[19] | Not target outcome: Multivariable adjusted HR for stroke |
| Zhao,X 2008 [20] | TyG index not measure |

Reference:

[1] Y. Zhao, H. Sun, W. Zhang, Y. Xi, X. Shi, Y. Yang, J. Lu, M. Zhang, L. Sun, and D. Hu, Elevated triglyceride–glucose index predicts risk of incident ischaemic stroke: The Rural Chinese cohort study. Diabetes and Metabolism 47 (2021).

[2] X. Zhao, Y. Wang, R. Chen, J. Li, J. Zhou, C. Liu, P. Zhou, Z. Sheng, Y. Chen, L. Song, H. Zhao, and H. Yan, Triglyceride glucose index combined with plaque characteristics as a novel biomarker for cardiovascular outcomes after percutaneous coronary intervention in ST-elevated myocardial infarction patients: an intravascular optical coherence tomography study. Cardiovascular Diabetology 20 (2021).

[3] A. Wang, X. Tian, Y. Zuo, S. Chen, X. Meng, S. Wu, and Y. Wang, Change in triglyceride-glucose index predicts the risk of cardiovascular disease in the general population: a prospective cohort study. Cardiovasc Diabetol 20 (2021) 113.

[4] K.W. Nam, M.K. Kang, H.Y. Jeong, T.J. Kim, E.J. Lee, J. Bae, K. Jeon, K.H. Jung, S.B. Ko, and B.W. Yoon, Triglyceride-glucose index is associated with early neurological deterioration in single subcortical infarction: Early prognosis in single subcortical infarctions. Int J Stroke (2021) 1747493020984069.

[5] K.W. Nam, H.M. Kwon, and Y.S. Lee, High triglyceride-glucose index is associated with early recurrent ischemic lesion in acute ischemic stroke. Scientific Reports 11 (2021).

[6] M. Lee, C.H. Kim, Y. Kim, M.U. Jang, H.J. Mo, S.H. Lee, J.S. Lim, K.H. Yu, B.C. Lee, and M.S. Oh, High Triglyceride Glucose Index Is Associated with Poor Outcomes in Ischemic Stroke Patients after Reperfusion Therapy. Cerebrovascular Diseases (2021).

[7] Z. Hou, Y. Pan, Y. Yang, X. Yang, X. Xiang, Y. Wang, Z. Li, X. Zhao, H. Li, X. Meng, and Y. Wang, An Analysis of the Potential Relationship of Triglyceride Glucose and Body Mass Index With Stroke Prognosis. Frontiers in Neurology 12 (2021).

[8] Y. Chang, C.K. Kim, M.-K. Kim, W.-K. Seo, and K. Oh, Insulin resistance is associated with poor functional outcome after acute ischemic stroke in non-diabetic patients. Scientific Reports 11 (2021).

[9] Y. Zhou, Y. Pan, H. Yan, Y. Wang, Z. Li, X. Zhao, H. Li, X. Meng, C. Wang, L. Liu, and Y. Wang, Triglyceride Glucose Index and Prognosis of Patients With Ischemic Stroke. Frontiers in Neurology 11 (2020).

[10] B. Zhang, L. Liu, H. Ruan, Q. Zhu, D. Yu, Y. Yang, X. Men, and Z. Lu, Triglyceride-Glucose Index Linked to Hospital Mortality in Critically Ill Stroke: An Observational Multicentre Study on eICU Database. Frontiers in Medicine 7 (2020).

[11] W. Shi, L. Xing, L. Jing, Y. Tian, H. Yan, Q. Sun, D. Dai, L. Shi, and S. Liu, Value of triglyceride-glucose index for the estimation of ischemic stroke risk: Insights from a general population. Nutrition, Metabolism and Cardiovascular Diseases 30 (2020) 245-253.

[12] X. Ma, L. Dong, Q. Shao, Y. Cheng, S. Lv, Y. Sun, H. Shen, Z. Wang, Y. Zhou, and X. Liu, Triglyceride glucose index for predicting cardiovascular outcomes after percutaneous coronary intervention in patients with type 2 diabetes mellitus and acute coronary syndrome. Cardiovascular Diabetology 19 (2020).

[13] H. Chiu, H.J. Tsai, J.C. Huang, P.Y. Wu, W.H. Hsu, M.Y. Lee, and S.C. Chen, Associations between triglyceride-glucose index and micro-and macro-angiopathies in type 2 diabetes mellitus. Nutrients 12 (2020).

[14] W.Y. Su, S.C. Chen, Y.T. Huang, J.C. Huang, P.Y. Wu, W.H. Hsu, and M.Y. Lee, Comparison of the effects of fasting glucose, hemoglobin a1c, and triglyceride–glucose index on cardiovascular events in type 2 diabetes mellitus. Nutrients 11 (2019).

[15] R.-C. Li, W.-D. Xu, Y.-L. Lei, T. Bao, H.-W. Yang, W.-X. Huang, and H.-R. Tang, The risk of stroke and associated risk factors in a health examination population A cross-sectional study. Medicine 98 (2019).

[16] K.B. Won, G.M. Park, S.E. Lee, I.J. Cho, H.C. Kim, B.K. Lee, and H.J. Chang, Relationship of insulin resistance estimated by triglyceride glucose index to arterial stiffness. Lipids in Health and Disease 17 (2018).

[17] K.B. Won, H.C. Kim, B.K. Lee, and H.J. Chang, Association between insulin resistance estimated by triglyceride glucose index and arterial stiffness. European Heart Journal 39 (2018) 119.

[18] J.L. Jin, Y.X. Cao, L.G. Wu, X.D. You, Y.L. Guo, N.Q. Wu, C.G. Zhu, Y. Gao, Q.T. Dong, H.W. Zhang, D. Sun, G. Liu, Q. Dong, and J.J. Li, Triglyceride glucose index for predicting cardiovascular outcomes in patients with coronary artery disease. Journal of Thoracic Disease 10 (2018) 6137-6146.

[19] L. Sánchez-Iñigo, D. Navarro-González, A. Fernández-Montero, J. Pastrana-Delgado, and J.A. Martínez, Risk of incident ischemic stroke according to the metabolic health and obesity states in the Vascular-Metabolic CUN cohort. International Journal of Stroke 12 (2017) 187-191.

[20] X. Zhao, S. Jiang, and Y. Tan, Insulin resistance and occurrence and prognosis of ischemic stroke: a non-randomized concurrent control and intra-group comparison. Neural regeneration research 3 (2008) 324‐328.

**Figure S1:**  Plot of the Leave-one out analysis


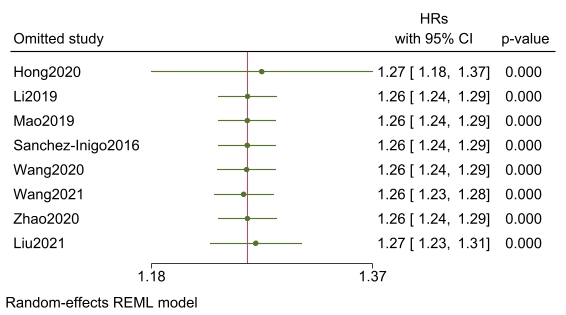

Supplement: Supplementary file 1 [file Data_Sheet_1.ZIP › Supplement 1.docx]
